# Health care worker burnout after the first wave of the coronavirus disease 2019 (COVID‐19) pandemic in Japan

**DOI:** 10.1002/1348-9585.12247

**Published:** 2021-08-10

**Authors:** Takahiro Matsuo, Fumika Taki, Daiki Kobayashi, Torahiko Jinta, Chiharu Suzuki, Akiko Ayabe, Fumie Sakamoto, Kazuyo Kitaoka, Yuki Uehara, Nobuyoshi Mori, Tsuguya Fukui

**Affiliations:** ^1^ Department of Infectious Diseases St. Luke’s International Hospital Tokyo Japan; ^2^ Department of Nephrology St. Luke’s International Hospital Tokyo Japan; ^3^ Division of Health Care Administration St. Luke’s International Hospital Tokyo Japan; ^4^ Department of General Internal Medicine St. Luke’s International Hospital Tokyo Japan; ^5^ Graduate School of Public Health St. Luke’s International University Tokyo Japan; ^6^ Department of Respiratory Medicine St. Luke’s International Hospital Tokyo Japan; ^7^ Department of Nursing St. Luke’s International Hospital Tokyo Japan; ^8^ Quality Improvement Center St. Luke's International Hospital Tokyo Japan; ^9^ Faculty of Health Sciences Komatsu University Ishikawa Japan; ^10^ Department of Clinical Laboratory Center St. Luke’s International Hospital Tokyo Japan

**Keywords:** burnout, COVID‐19, Maslach burnout inventory

## Abstract

**Objectives:**

To determine the prevalence of burnout according to job category after the first wave of COVID‐19 in Japan and to explore its association with certain factors.

**Methods:**

An online cross‐sectional survey of health care workers (HCWs) from June 15 to July 6, 2020, was conducted at a tertiary hospital in Tokyo, Japan. Demographic characteristics, results of the Japanese version of the Maslach Burnout Inventory‐General Survey, types of anxiety and stress, changes in life and work after the peak of the pandemic, and types of support aimed at reducing the physical or mental burden, were determined.

**Results:**

Of 672 HCWs, 149 (22.6%) met the overall burnout criteria. Burnout was more prevalent in women (OR, 3.11; 95% CI, 1.45‐6.67, *P* = .003), anxiety due to unfamiliarity with personal protective equipment (PPE) (OR, 1.98; 95% CI, 1.20‐3.27, *P* = .007), and decreased sleep duration (OR, 1.96; 95% CI, 1.20‐3.20, *P* = .008). Conversely, participants who felt that the delivery of COVID‐19‐related information (OR, .608; 95% CI, .371‐.996, *P* = .048) and PPE education opportunities (OR, .484; 95% CI, .236‐.993, *P* = .048) and messages of encouragement at the workplace (OR, .584; 95% CI, .352‐.969; *p* = .037) was helpful experienced less burnout.

**Conclusions:**

There is a need to focus on the above factors to maintain the mental health of HCWs. The delivery of COVID‐19‐related information and educational interventions for PPE and messages of encouragement at the workplace may be needed to reduce the mental burden.

## 1 INTRODUCTION

Coronavirus disease 2019 (COVID‐19) is an infection caused by severe acute respiratory syndrome coronavirus 2 (SARS‐CoV‐2). First detected in December 2019.^1^ COVID‐19 remains an ongoing international public health emergency. As of February 2, 2021, COVID‐19 had infected more than 103 930 000 individuals and killed more than 2 247 000 worldwide.^2^ These emergencies have caused the collapse of health systems across numerous countries,^3^ including shortages of beds and frontline health care workers (HCWs). In addition, many infections among HCWs themselves have been reported, resulting in the deaths of more than 3000 HCWs in the United States.^4^

Apart from the physical burden induced by increased workloads, longer working hours, decreased sleep duration, and irregular eating habits,^5^, ^6^, ^7^ several psychological effects have been noted in HCWs, including depression, anxiety, insomnia, and burnout.^8^ HCWs are routinely exposed to pressure related to social expectations, experiences with patients having unfortunate outcomes, adjustments caused by personal protective equipment (PPE) shortages, and involvement in emotionally and ethically fraught resource‐allocation decisions.^5^ Given that burnout among HCWs may promote increased turnover, medical facilities may experience periods of insufficient staffing.

Several recent studies have examined factors that contribute to burnout among HCWs during the COVID‐19 pandemic.^9^, ^10^, ^11^ Our previous study^12^ found that more than 40% of nurses and 30% of radiological technologists and pharmacists who had direct contact with patients with COVID‐19 in our institute satisfied the criteria for burnout. Although a number of studies have reported on burnout during the peak of the pandemic, only a few have explored its prevalence after the pandemic had temporarily subsided. Furthermore, few studies have analyzed the peak of the pandemic after it had temporarily subsided and determined which changes or support were associated with the physical and mental burden during the pandemic.

Therefore, the current study aimed to examine the prevalence of burnout after the pandemic had temporarily subsided and explore its correlation with changes in life and work after the peak of pandemic and types of support in order to maintain their mental health during subsequent pandemics.

## 2 METHODS

### 2.1 Study design and participants

**FIGURE 1:**
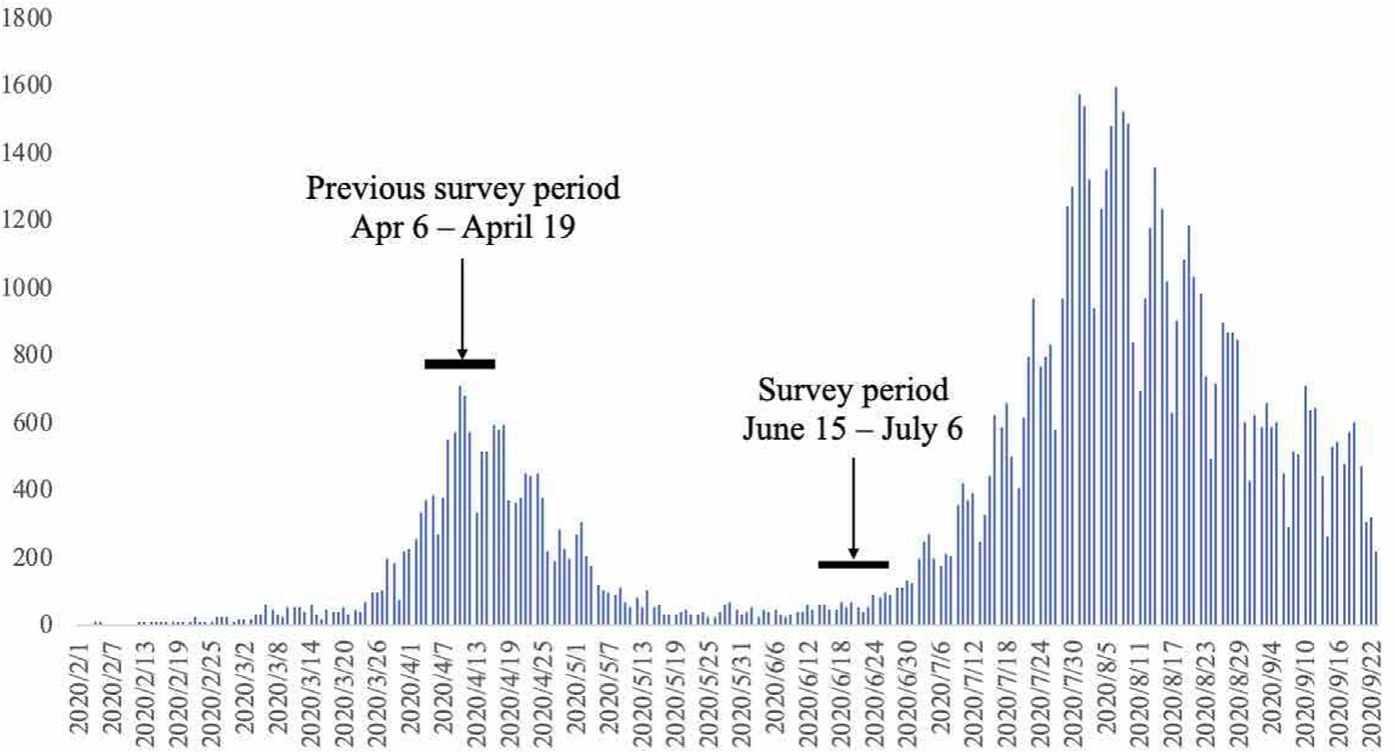
Number of daily confirmed positive COVID‐19 cases in Japan and the date of the survey

An online cross‐sectional survey among HCWs (n = 1672) was conducted between June 15 and July 6, 2020––after the first wave of the pandemic (Figure 1)––at St. Luke’s International Hospital, a tertiary hospital in Tokyo, Japan with 525 standard beds and 25 intensive care unit beds. Our hospital has treated more than 220 confirmed and 350 suspected patients, accounting for 3.2% of the 5587 confirmed patients in Tokyo by June 15, 2020. All HCWs, including physicians, nurses, laboratory medical technologists, radiological technologists, pharmacists, clinical engineering technologists, physical therapists, registered dieticians, medical clerks, and receptionists, were included. Information regarding the web survey’s Uniform Resource Locator was provided. The web‐based survey was generated using Questant (Macromill, Inc, Japan), a cloud‐based survey development application. Participants were given 3 weeks to respond.

### 2.2 Questionnaire

The survey solicited responses regarding participants’ demographics (age and gender), professional history (job category and years of experience), working environment (mean weekly working hours, days off per month, frequency of direct contact with patients with COVID‐19 per week, and frequency of COVID‐19‐related work per week), and life environment (mean sleep duration per day and days off per month). The job category of each participant was identified as one of the following: physician, nurse, laboratory medical technologist, radiological technologist, pharmacist, clinical engineering technologist, physical therapist, registered dietician, medical clerk, and receptionist. Questions were answered based on the average over the past month. Frontline workers were defined as participants who had direct contact with COVID‐19 patients at least once a week. Involvement in COVID‐19‐related work included not only direct contact with patients with COVID‐19 but also bed control, infection control guidance, responding to inquiries from local medical facilities and patients, paperwork, and reporting to the administration and local public health center. Other potential factors, such as dropout intention, types of anxiety perceived, changes in life and work after the peak of the pandemic, and types of changes or support needed to reduce physical or mental burden, were also investigated. The survey items related to types of anxiety perceived and types of changes or support needed to reduce physical or mental burden were discussed and categorized by the COVID‐19 mental support team, which consisted of 13 members from our hospital. In this series of work, survey items used in the previous survey (April, 2020)^12^ and those freely mentioned at the time were used as references. Working environmental improvement included clarification of COVID‐19 zones; creation of special floors for patients with COVID‐19 in intensive care units and general wards; introduction of transparent partitions in reception areas and wards to prevent droplet infection; and securing items, such as surgical masks, N95, and PPE. Staff adjustment included closing some wards and departments, increasing support to other departments, and changing working shifts and locations. Messages of encouragement at the workplace included positive messages from coworkers or leaders. Delivery of COVID‐19‐related information specifically indicated that the infection control nurse delivered a 15‐minute online update related to COVID‐19 to all staff once a week. PPE education opportunities indicated that infection control nurses developed an in‐hospital manual of attachment and doffing procedures, while infectious disease physicians, respiratory physicians, and chief medical residents created either face‐to‐face or online training opportunities in wards for HCWs who might have direct contact with patients with COVID‐19. Support from society included encouraging letters from elementary schools or communities and food and snack deliveries from companies. Items were assessed using a 6‐point Likert scale ranging from “0” (*disagree*) to “5” (*agree*). During analysis, a rating of “4” and “5” indicated “*agree*”. Changes in life and work after the peak of the pandemic was assessed using a 5‐point Likert scale including “*deteriorated*,” “*relatively deteriorated*,” “*no change*,” “*relatively improved*,” and “*improved*”. During analysis, a rating of “*deteriorated*” and “*relatively deteriorated*” indicated “*deteriorated*,” while “*relatively improved*” and “*improved*” indicated “*improved*.”

### 2.3 Outcome

The primary outcome was overall burnout prevalence among HCWs, which was assessed using the Japanese version of the Maslach Burnout Inventory‐General Survey (MBI‐GS),^13^ a validated version of the MBI that is currently considered the gold standard for measuring burnout.^14^ This 16‐item questionnaire contains three subscales that evaluate the three generally accepted major domains: emotional exhaustion, cynicism (depersonalization), and professional efficacy (personal accomplishment). Each of the 16 items was scored on a 7‐point scale ranging from “0” (*never*) to “6” (*every day*), while total scores for each subscale were divided by the number of items in each subscale. Based on a previous survey of the Japanese population, the primary criteria for burnout used herein was high exhaustion plus one of either high cynicism or low professional efficacy, which has been considered the most appropriate and reliable indicator of burnout among the general population.^15^ Essentially, we included those who satisfied the exhaustion plus one criterion originally introduced by Brenninkmeijer and Van Yperen who viewed burnout as a binary outcome.^16^ Cut‐off points reported by Kalimo et al^17^ specifically >3.5 for exhaustion and cynicism and <2.5 for professional efficacy, were used.

### 2.4 Statistical analyses

Initially, baseline characteristics of participants who did and did not have burnout were compared using the chi‐square difference tests and Mann‐Whitney U test for categorical and continuous variables respectively. We performed multivariable logistic regression, controlling for potential covariates including age, gender, and type of occupation, defined as clinically important variables or those with a *P*‐value < .05 on bivariate analyses. All analyses were performed using SPSS version 19.0 software (IBM Japan, Tokyo, Japan) and STATA 11 (STATA Corp., College Station, TX, USA), with a two‐tailed *P* value of <.05 indicating significance.

### 2.5 Ethical considerations

Requests for research cooperation were sent to participants via their hospital‐account email address, with questionnaire completion implying consent. The cover letter explained that participation would be voluntary and that the participants’ responses would remain anonymous. The questionnaire system on the web was not linked to the original email account.

This study was approved by the Institutional Review Board of St. Luke’s International Hospital in Tokyo, Japan (Number: 20‐R051).

## 3 RESULTS

**FIGURE 2:**
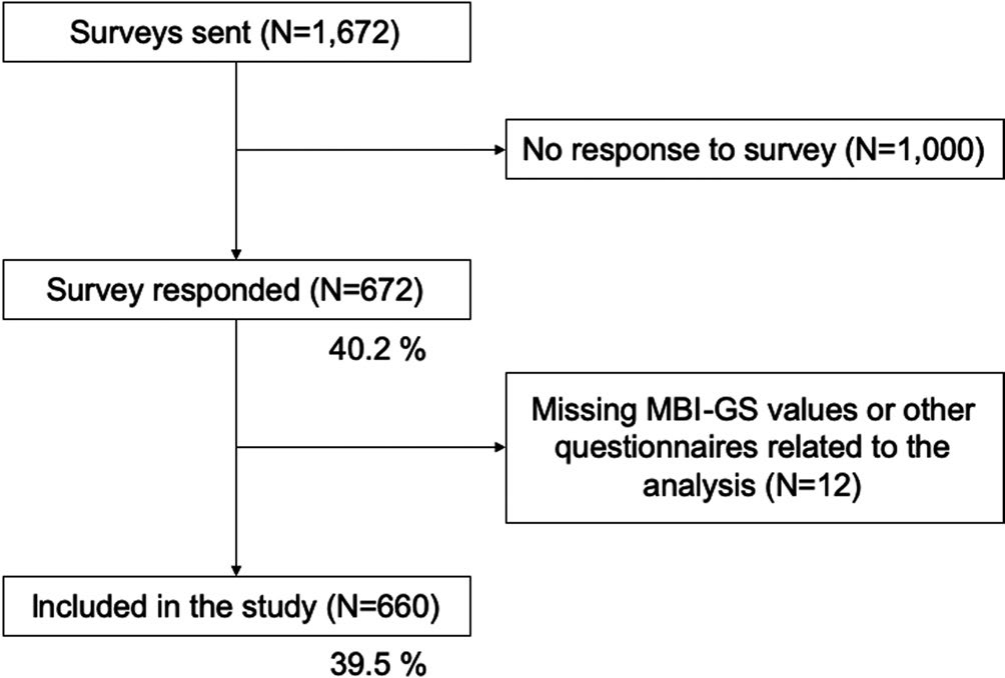
Flow chart of the sample selection process

Among the 1672 HCWs, 672 (40.2%) responded to the survey, among whom 12 were excluded from the survey because of missing values in the MBI‐GS and other questionnaires related to the analysis (Figure 2). The final sample included 660 respondents, 77.7% of whom were women (n = 513). The overall burnout prevalence was 22.6% (n = 149).

### 3.1 Comparison between participants with and without burnout

Table 1 compares participants with and without burnout based on their demographics. Compared to the non‐burnout group, the burnout group had a significantly higher percentage of participants who were women (92.6% vs 73.4%; *P* = .001), were younger (21‐30 years old) (51.0% vs 31.5%; *P* = .001), had fewer years of experience (1‐2 years) (29.5% vs 18.6%, *P* = .001), and had dropout intentions (77.2% vs 22.7%; *P* = .001). Interestingly, no significant differences in the percentage of direct contacts with patients with COVID‐19, involvement with COVID‐19‐related work, working hours per week, sleep duration per day, and number of days off per month was observed between both groups.

**TABLE 1:** Comparison of demographic characteristics, changes after the peak of pandemic (March‐April 2020), and factors perceived to be beneficial in reducing physical or mental burden between participants with and without burnout

	Burnout (+)	Burnout (−)	Overall	*P*
	n = 149	n = 511	N = 660	
Women, n (%)	138 (92.6)	375 (73.4)	513 (77.7)	.**001**
Age, n (%), years				.**001**
21‐30	76 (51.0)	161 (31.5)	237 (35.9)	
31‐40	42 (28.2)	161 (31.5)	203 (30.8)	
41‐50	24 (16.1)	114 (22.3)	138 (20.9)	
51‐60	7 (4.7)	63 (12.3)	70 (10.6)	
>60	0 (0.0)	12 (2.3)	12 (1.8)	
Occupation, n (%)				.**001**
Physician	9 (6.0)	83 (16.2)	92 (13.9)	
Nurse	109 (73.1)	262 (51.2)	371 (56.2)	
Laboratory medical technologist	8 (5.4)	45 (8.8)	53 (3.0)	
Radiological technologist	5 (3.4)	17 (3.3)	22 (3.3)	
Pharmacist	6 (4.0)	14 (2.7)	20 (3.0)	
Clinical engineering technologist	2 (1.3)	10 (2.0)	12 (1.8)	
Physical therapist	0 (0.0)	13 (2.5)	13 (2.0)	
Registered dietician	0 (0.0)	7 (1.4)	7 (1.1)	
Medical clerk	8 (5.4)	48 (9.4)	56 (8.5)	
Receptionist	1 (0.7)	8 (1.6)	9 (1.4)	
Experience, n (%), years				.**001**
1‐2	44 (29.5)	95 (18.6)	136 (20.6)	
3‐6	38 (25.5)	98 (19.2)	139 (21.1)	
7<	67 (45.0)	318 (62.2)	385 (58.3)	
Frontline workers, n (%)	49 (32.9)	156 (30.5)	205 (31.1)	.615
Involvement with COVID‐19‐related work, n (%)	66 (44.3)	243 (47.6)	309 (46.8)	.514
Working hours per week, median (IQR), hours	39 [44‐55]	40 [40‐55]	42 [40‐55]	.992
Sleep duration per day, median (IQR), hours	6.43 [5.86‐7.00]	6.29 [5.86‐7.00]	6.28 [5.85‐7.00]	.984
Days off per month, median (IQR), days	8.0 [8.0‐10.0]	8.0 [8.0‐10.0]	8.0 [8.0‐10.0]	.231
Dropout intentions, n (%)	115 (77.2)	116 (22.7)	429 (65.0)	.**001**
Types of anxiety or stress perceived, n (%)				
Getting COVID‐19	126 (84.6)	385 (75.3)	511 (77.4)	.**019**
Transmission to family members	117 (78.5)	371 (72.6)	488 (73.9)	.168
Transmission to coworkers and friends	122 (81.9)	391 (76.5)	513 (77.7)	.181
Transmission to patients	113 (75.8)	346 (67.7)	459 (69.5)	.068
Unfamiliarity with PPE	35 (23.5)	74 (14.5)	109 (16.5)	.**012**
Lack of daily necessities	74 (49.7)	188 (36.8)	262 (39.7)	.**006**
Childcare	24 (16.1)	121 (23.7)	145 (22.0)	.056
The need to refrain from going out	137 (91.9)	431 (84.3)	568 (86.1)	.**022**
Decreased income	57 (38.3)	152 (29.7)	209 (31.7)	.057
Changes compared to the peak of pandemic (March‐April 2020), n (%)				
Working hours				.**009**
Deteriorated	44 (29.5)	93 (18.2)	137 (20.8)	
Improved	40 (26.8)	175 (34.2)	215 (32.6)	
No change	65 (43.6)	243 (47.6)	308 (46.7)	
Workload				.**007**
Deteriorated	62 (41.6)	145 (28.4)	207 (31.4)	
Improved	36 (24.2)	170 (33.3)	206 (31.2)	
No change	51 (34.2)	196 (38.4)	247 (37.4)	
Healthy eating habits				.**001**
Deteriorated	52 (34.9)	94 (18.4)	146 (22.1)	
Improved	25 (16.8)	89 (17.4)	114 (17.3)	
No change	72 (48.3)	328 (64.2)	400 (60.6)	
Sleep length				.**001**
Deteriorated	57 (38.3)	96 (18.8)	153 (23.2)	
Improved	11 (7.4)	69 (13.5)	80 (12.1)	
No change	81 (54.4)	346 (67.7)	427 (64.7)	
Exercise				.096
Deteriorated	65 (43.6)	174 (34.1)	239 (36.2)	
Improved	16 (10.7)	70 (13.7)	86 (13.0)	
No change	68 (45.6)	267 (52.3)	335 (50.8)	
Excessive caffeine/alcohol				.164
Deteriorated	34 (22.8)	89 (17.4)	123 (18.6)	
Improved	15 (10.1)	39 (7.6)	54 (8.2)	
No change	100 (67.1)	383 (75.0)	483 (73.2)	
Relaxation time				.059
Deteriorated	73 (49.0)	197 (38.6)	270 (40.9)	
Improved	25 (16.8)	90 (17.6)	115 (17.4)	
No change	51 (34.2)	224 (43.8)	275 (41.7)	
What factors that helped reduce physical or mental burden, n (%)				
Convergence of infection	112 (75.2)	373 (73.0)	485 (73.5)	.673
Working environmental improvement	62 (41.6)	271 (53.0)	333 (50.5)	.**016**
Staffing adjustment	38 (25.5)	152 (29.7)	190 (28.8)	.355
Getting used to dealing	42 (28.2)	187 (36.6)	229 (34.7)	.063
Messages of encouragement at the workplace	29 (19.5)	151 (29.5)	180 (27.3)	.**016**
Delivery of COVID‐19‐related information	36 (24.2)	218 (42.7)	254 (38.5)	.**001**
PPE education opportunities	13 (8.7)	120 (23.5)	133 (20.2)	.**001**
Support for childcare	1 (0.7)	16 (3.1)	17 (2.6)	.139
Support from family members	54 (36.2)	190 (37.2)	244 (37.0)	.848
Support from society	61 (40.9)	244 (47.7)	305 (46.2)	.161
Mental support	3 (2.0)	12 (2.3)	15 (2.3)	1.000
Support for accommodation	5 (3.4)	22 (4.3)	27 (4.1)	.814
Support for special leave	13 (8.7)	39 (7.6)	52 (7.9)	.729
Implementing online conferencing tools	27 (18.1)	149 (29.2)	176 (26.7)	.**008**

(+), participants with burnout; (−), participants without burnout; IQR, interquartile range; COVID‐19, Coronavirus Disease 2019; PPE, personal protective equipment.

Values with *P* < .05 are in bold.

**FIGURE 3:**
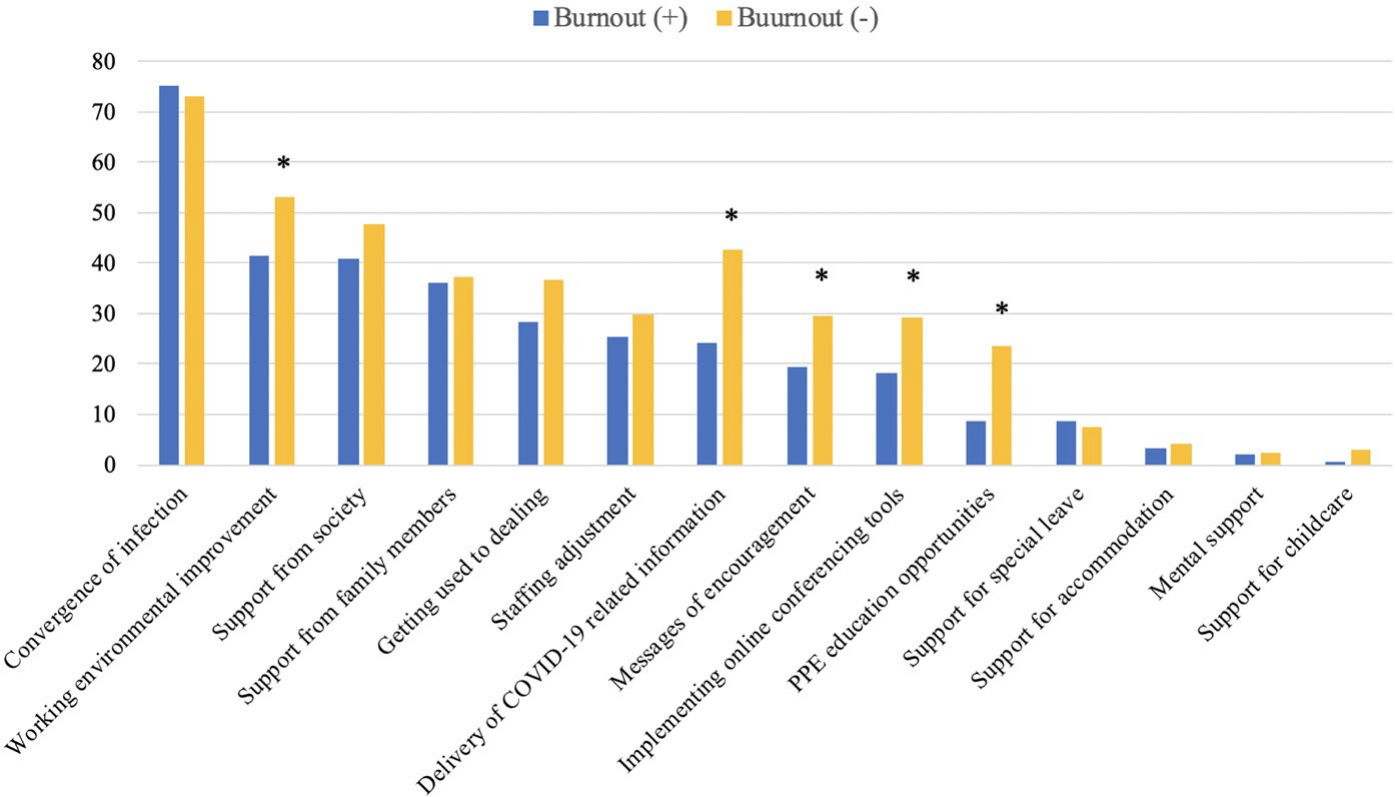
Changes or support that helped reduce the physical and mental burden

Participants exhibiting burnout reported more anxiety or stress related to acquiring COVID‐19 (84.6% vs 15.4%; *P* = .001), unfamiliarity with PPE (23.5% vs 14.5%; *P* = .012), lack of daily necessities (49.7% vs 36.8%; *P* = .006), and the need to refrain from going out (91.9% vs 84.3%; *P* = .022), as well as increased working hours (*P* = .009), increased workloads (*P* = .007), unhealthy eating habits (*P* = .001), and insufficient sleep duration (*P* = .001) after the peak of the pandemic. Conversely, those without burnout reported that improvement in working environmental (53.0% vs 41.6%; *P* = .016), messages of encouragement at the workplace (29.5% vs 19.5%; *P* = .016), delivery of COVID‐19‐related information (42.7% vs 24.2%; *P* = .001), PPE education opportunities (23.5% vs 8.7%; *P* = .001), and implementing online conferencing tools (29.2% vs 18.1%; *P* = .008) helped reduce their physical and mental burden (Figure 3). In the subgroup analysis stratified by occupation, there was no difference in the prevalence of burnout by occupation between frontline and non‐frontline workers (Supplement 1).

### 3.2 Adjusted odds ratio for the multivariable model of burnout

The results of the multivariable analyses are presented in Table 2. After adjusting for potential covariables, burnout was more prevalent in participants who were women (OR, 3.11; 95% CI, 1.45‐6.67; *P* = .003), and had heightened anxiety due to unfamiliarity with PPE (OR, 1.98; 95% CI, 1.20‐3.27; *P* = .007). Moreover, those whose sleep duration had “deteriorated” showed a significantly higher burnout prevalence than those who exhibited “no change” in sleep duration (OR, 1.96; 95% CI, 1.20‐3.20; *P* = .008). Conversely, participants who reported that PPE education opportunities (OR, 0.484; 95% CI, 0.236‐0.993; *P* = .048) and messages of encouragement at the workplace (OR, 0.584; 95% CI, 0.352‐0.969; *P* = .007) helped reduce their physical and mental burden had less burnout.

**TABLE 2:** Factors associated with burnout (multivariable analysis using the logistic regression model)

	Adjusted OR	*P*	95% CI
Women	3.11	.**003**	1.45‐6.67
Age			
20	2.45	.103	0.835‐7.17
30	1.58	.328	0.633‐3.93
40	1.62	.316	0.630‐4.18
50	Reference	.405	
Type of occupation			
Physician	Reference	.130	
Nurse	2.07	.114	0.840‐5.10
Others	1.36	.517	0.541‐3.40
Years of experience			
1‐2	1.23	.563	0.614‐2.45
3‐6	0.91	.797	0.443‐1.87
≥7	Reference	.578	
Frontline workers	1.04	.865	0.670‐1.61
Working hours per week	1.00	.767	0.992‐1.01
Sleep duration per day	0.970	.787	0.776‐1.21
Working hours			
Deteriorated	1.26	.514	0.667‐2.25
Improved	1.18	.614	0.622‐2.23
No change	Reference	.757	
Workload			
Deteriorated	1.18	.573	0.663‐2.10
Improved	0.83	.592	0.413‐1.66
No change	Reference	.640	
Sleep length			
Deteriorated	1.96	.**008**	1.20‐3.20
Improved	0.766	.503	0.351‐1.67
No change	Reference	.019	
Anxiety due to unfamiliarity with PPE	1.98	.**007**	1.20‐3.27
Messages of encouragement at the workplace	0.584	.**007**	0.352‐0.969
Delivery of COVID‐19 related information	0.684	.149	0.409‐1.15
PPE education opportunities	0.484	.**048**	0.236‐0.993

OR, odds ratio; PPE, personal protective equipment; COVID‐19, Coronavirus Disease 2019.

Values with *P* < .05 are in bold.

## 4 DISCUSSION

This has been the first study in Japan to examine the prevalence of burnout among HCWs after the peak of the COVID‐19 pandemic had temporarily subsided and identify which changes or support helped to reduce the physical and mental burden during the pandemic. Our results found that the prevalence of burnout among HCWs at our institute was 22.6% in June 2020, when the number of patients positive for COVID‐19 decreased to less than 50 cases per day throughout Japan.

Previous studies among HCWs in Japan and other countries during the COVID‐19 pandemic reported high overall burnout prevalence, ^9^, ^18^, ^19^, ^20^, ^21^, ^22^, ^23^ as well as our previous study conducted during the peak of the first wave (April 2020) among participants from the same institute (31.4%).^12^ Also, several studies have also reported a higher prevalence of burnout compared to pre‐pandemic levels.^24^, ^25^ These studies suggested that the prevalence of burnout is increasing as a result of the pandemic. Previous studies have also reported burnout as a serious problem in the COVID‐19 pandemic among non‐HCWs, such as teachers,^26^ security forces,^27^ and managers. Although HCWs are often reported to have a higher psychological burden than non‐HCWs,^28^, ^29^ the number of patients with COVD‐19 in each region, the timing of the survey, and the type of the questionnaire and cut‐off point of the scale differ among studies. Therefore, it should be noted that the prevalence of burnout in this study cannot be directly compared with other studies.

### 4.1 Associating factors of burnout after first wave of the pandemic

Logistic regression analysis identified female gender, deteriorated sleep length, and anxiety due to unfamiliarity with PPE as positively associating factors of burnout. Also, although not identified as a significant factor in the multivariable analysis, younger and less experienced HCWs had a higher prevalence of burnout. Similar trends had been observed in our previous study conducted during the peak of the pandemic.^12^

Female gender^15^ or fewer years of experience^30^ have generally been considered risk factors for burnout. During the COVID‐19 pandemic, it has been reported that female HCWs with fewer years of experience were more likely to experience anxiety, depression, and fear,^22^, ^23^ which has been linked to burnout.^18^ Burnout is defined as a state of psychological, emotional, and physical stress due to prolonged exposure to occupational stress.^31^ It is easy to imagine that they will be under a lot of strain and may suffer from burnout during this pandemic. Indeed, studies before COVID‐19 have shown that HCWs with fewer years of experience are less resilient than those with more experience,^32^ women experience higher emotional exhaustion than men,^33^ and work‐family conflict is associated with burnout in women,^34^ all of which may still hold true during the COVID‐19 pandemic.

Our finding of an association between anxiety due to unfamiliarity with PPE and burnout was consistent with that presented in our previous report conducted in April 2020.^12^ Studies have shown that some HCWs without proper training use PPE incorrectly.^35^ Accordingly, this lack of specialized training has been found to be a risk factor for burnout. Given that gown doffing can be particularly challenging,^36^ providing frequent reminders to avoid touching the eyes and face^37^ and learning proper doffing techniques are essential. Confidence in the use of PPE and less fear in becoming infected may be considered factors preventing burnout.

Notably, despite both groups having the similar average daily sleep duration, participants who perceived deterioration in sleep duration after the peak of the pandemic experienced more burnout. The hypotheses for the result that participants perceived the deterioration of sleep duration even though sleep duration remained the same between the two groups are as follows: (i) Since the burnout group contained more participants with longer baseline sleep duration, the average sleep duration was similar as the non‐burnout group even though sleep duration was reduced. (ii) Despite the increase in mid‐waking reported in previous studies, participants may have responded to the sleep duration questionnaire as time from bedtime to waking without including mid‐waking, and responded to the change in sleep duration questionnaire as “worsened.” Despite the temporary decrease in the number of patients with COVID‐19, our study results may suggest that sleep deprivation may be associated with burnout. It should be noted that since this study is not a longitudinal study, whether acute change is due to the pandemic or other potential factors is unknown. In the midst of a pandemic, adjusting staffing schedules and reducing overtime hours are important despite potentially causing excessive workloads. Providing education regarding quality sleep practices and routines may also help promote proper sleep.^38^ Considering that burnout often remains unrecognized by those experiencing the same and is not easily recognized by others, sleep deprivation may be used as an early indicator of burnout during the COVID‐19 pandemic.

### 4.2 Frontline vs non‐frontline HCW

From another point of view, our results showed that the incidence of HCW burnout were the same regardless of whether they were frontline or non‐frontline workers. Burnout could be a problem not only for frontline HCWs, who face an increased burden from COVID‐19 patient care itself, but also for non‐frontline HCWs who have anxiety and stress caused by new policy procedures and the possibility of identifying an infected person at any time in non‐frontline settings. Moreover, the fact that even asymptomatic patients can be infectious^39^ may contribute to anxiety or fear among all HCWs. Other factors that may contribute to the physical and emotional burden on both frontline and non‐frontline HCWs include recent subjective symptom questioning of all patients, thoroughness of ventilation and disinfection, increased telephone inquiries, and stress outside the hospital (eg, refraining from going out and social distancing).

### 4.3 Working hours and workloads after the peak of the pandemic

Although an objective assessment cannot be made based on the cross‐sectional study, 207 respondents (31.4%) reported that their working hours had worsened compared to the peak period.

A sub‐analysis of the three groups of occupations (physicians, nurses, and others) showed that the percentage of nurses who reported that their working hours had worsened was significantly higher than that of physicians and other occupations (16.3% vs 25.3% vs 14.2%, *P* = .04). In terms of changes in workload, although not significant, the percentage of nurses who reported that their workload had increased tended to be higher than that of other occupations (34.2 vs 27.7%, *P* = .072). These results suggest that nurses may be experiencing an increase in workload and working hours, despite all but physicians work in shifts. Regardless of the peak of the pandemic, it is possible that there might be an increase in personal protective equipment response to patients with fever or an increase in workload per person due to a decrease in staff outside the department in charge of COVID‐19.

### 4.4 Importance of PPE and COVID‐19 education

Finally, the current study identified which among the interventions introduced by the hospital in supporting its staff over the past 2 months were associated with less burnout. Our findings showed that participants who perceived that PPE education opportunities and messages of encouragement at the workplace were effective had less burnout.

Since the beginning of the pandemic, infection control nurses in our hospital have widely disseminated COVID‐19‐related information, introduced preventive measures against COVID‐19 online, and provided entire hospital education. Moreover, infectious disease physicians, respiratory physicians, chief medical residents, and infection control nurses have visited the wards in person or online to provide guidance on the use of PPE attachment and doffing. When direct training is impossible because of social distancing limitations, online platforms, such as webinars or multimedia chatting, can be used to provide remote interactive training opportunities.^40^ Although it is true that direct comparison could not be made, compared to our previous study in April 2020,^12^ the current study observed a dramatic decrease in the percentage of individuals experiencing anxiety due to unfamiliarity of PPE (from 80.1% [250/312] to 16.5% [109/660]), suggesting that our educational interventions on the proper use of PPE may have been able to alleviate participants' fear or anxiety. These interventions can be implemented in any facility and may help prepare for future flare‐ups of the pandemic.

Moreover, it is noteworthy that our study revealed that encouraging messages in the workplace could be associating factor of less burnout. It could be essential for facility and team leaders to recognize the value of each other and work to create a collaborative work environment, which may lead to a reduction in burnout.^41^ Promoting ways to connect with colleagues and share successes, and delivering positive messages that emphasize appreciation for members' dedication and altruism may help members find joy in chaotic situations.^42^

### 4.5 Strengths and limitations

The strengths of the current study were centered in our ability to (i) investigate burnout prevalence once the pandemic had subsided, (ii) overcome the limitations of our previous study,^12^ which included only frontline HCWs over five professions, and expand the survey to include HCWs from over 10 professions who were both directly and indirectly involved with patients with COVID‐19, and (iii) assess which changes or supports were perceived to be effective in reducing physical and mental burden among those who did and did not experience burnout.

Nonetheless, the present study has several limitations. First, given that our study was conducted at a single institution, our results may not be generalizable to other countries or regions. Presuming that facilities accepting patients with COVID‐19 have similar problems, our findings may be used as reference for establishing strategies that maintain the mental health of HCWs. Second, the response rate was relatively low. Although this was a hospital‐wide survey focusing on different types of job categories, in a survey where responses are voluntary, there are several possible causal factors for those who chose not to participate in the survey. For example, people with symptoms of burnout may not have completed the survey because they were worried about being identified or did not have sufficient mental capacity to complete the survey, which could lead to underestimation. It is also possible that people with severe symptoms may have already quit their jobs and therefore may not be considered in the non‐responder bias. Third, the actual changes were not known in this cross‐sectional study because the questions about changes (eg, workload, sleep time) mentioned above are based on the subjective perceptions of individuals. Further longitudinal studies may enable to identify the relationship between the actual amount of each change and burnout.

Fourth, the long‐term impact of our results remains unclear considering that this study was conducted over a short period of time. During the MERS‐CoV outbreak, studies had reported persistent symptoms, including anxiety and anger, even after 4‐6 months of convergence.^43^ Long‐term psychological distress and post‐traumatic stress have also been reported after the SARS outbreak. Further studies should therefore focus on changes in burnout prevalence after interventions that mitigate their long‐term effects. Finally, participants in this survey were relatively younger, with the majority being women (77%). Broadening the age range and increasing the proportion of male responders in future studies could potentially yield new findings. There are few studies investigating the prevalence of burnout in other professions during the subsiding phase of the pandemic. Therefore, further longitudinal studies are needed to confirm the change in prevalence of burnout using the same questionnaire and comparing it with other professions during the pandemic transition.

Current predictions suggest that this pandemic will not be temporary and would instead likely continue for even a few years. Based on the findings obtained herein, future studies should investigate long‐term trends in burnout at different time points during future pandemics and apply ongoing interventions. Supporting the mental well‐being of HCWs and preventing burnout are essential for the provision of ongoing patient care, which would definitely contribute toward ending the COVID‐19 pandemic.

## 5 CONCLUSIONS

This has been the first study in Japan to explore the prevalence of burnout after the pandemic had subsided and its association with changes or support perceived to be effective. Our results found that 22.6% of HCWs who responded to our survey satisfied the criteria for burnout in June 2020. Accordingly, female sex, and anxiety due to unfamiliarity with PPE were identified as factors independently associated with burnout. Moreover, participants who reported that PPE education opportunities and messages of encouragement at the workplace helped reduce their physical and mental burden had less burnout.

## DISCLOSURES

*Approval of the research protocol*: This study was approved by the Institutional Review Board of St. Luke’s International Hospital in Tokyo, Japan (Number: 20‐R051). *Informed* *consent*: N/A. *Registry and the* *registration no*. *of the study/trial*: N/A. *Animal* *studies*: N/A. *Conflict of interests*: The authors declare no conflict of interests for this article.

## AUTHOR CONTRIBUTIONS

The manuscript was seen and approved by all the authors and is not under consideration elsewhere. All the authors contributed to the work in this report. T.M. wrote the initial draft of the manuscript. F.T. collected clinical data. T.M. and D.K. analyzed the data. T.J., C.S., A.A., F.S., K.K., Y.U., N.M., and T.F. supervised the writing of the manuscript.

## Supporting information

Table S1
