# Supplementary material for: Health care worker burnout after the first wave of the coronavirus disease 2019 (COVID‐19) pandemic in Japan
Source: J Occup Health. 2021 Aug 10;63(1):e12247. doi: 10.1002/1348-9585.12247 (PMC8354617; doi:10.1002/1348-9585.12247)
Supplement: Supplementary file 1 — Table S1 [file JOH2-63-e12247-s001.docx]

**Supplement 1. Subgroup analysis stratified by occupation**

| Occupation |  | *Burnout* (+) | *Burnout* (-) | *Overall* | *P value* |
| --- | --- | --- | --- | --- | --- |
| Physician | Frontline, n (%) | 5 (55.6) | 35 (42.2) | 40 (43.5) | 0.442 |
|  | Non-frontline, n (%) | 4 (44.4) | 48 (57.8) | 52 (56.5) |  |
| Nurse | Frontline, n (%) | 34 (31.2) | 86 (32.8) | 120 (32.2) | 0.760 |
|  | Non-frontline, n (%) | 75 (68.8) | 176 (67.2) | 251 (67.7) |  |
| Others | Frontline, n (%) | 10 (32.3) | 35 (21.1) | 45 (22.8) | 0.174 |
|  | Non-frontline, n (%) | 21 (67.7) | 131 (78.9) | 152 (77.2) |  |
